# Endocardite Infecciosa em Idosos: Características Distintas

**DOI:** 10.36660/abc.20201134

**Published:** 2021-10-06

**Authors:** Luiz Henrique Braga Lemos, Leonardo Ribeiro da Silva, Marcelo Goulart Correa, Wilma Golebiovski, Clara Weksler, Rafael Quaresma Garrido, Giovanna Ferraiuoli Barbosa, Cristiane da Cruz Lamas

**Affiliations:** 1 Universidade do Grande Rio Rio de JaneiroRJ Brasil Universidade do Grande Rio (Unigranrio), Rio de Janeiro, RJ – Brasil; 2 Instituto Nacional de Cardiologia Rio de JaneiroRJ Brasil Instituto Nacional de Cardiologia, Rio de Janeiro, RJ – Brasil; 3 Instituto Nacional de Infectologia Evandro Chagas Fiocruz Rio de JaneiroRJ Brasil Instituto Nacional de Infectologia Evandro Chagas, Fiocruz, Rio de Janeiro, RJ – Brasil; 4 Universidade do Estado do Rio de Janeiro Rio de JaneiroRJ Brasil Universidade do Estado do Rio de Janeiro, Rio de Janeiro, RJ – Brasil

**Keywords:** Endocardite, Idoso, Comorbidade, Mortalidade, Cirurgia Torácica

## Introdução

A endocardite infecciosa (EI) é uma doença grave, com mortalidade intra-hospitalar média de 20%.^1 - 3^ Apresenta incidência crescente, com destaque para o aumento de sua prevalência na população idosa.^4 - 9^ Em pacientes da terceira idade com EI, existem diferenças quanto a apresentação clínica, complicações, presença de comorbidades, abordagem terapêutica e mortalidade.^4 , 10 - 13^ Diretrizes de EI não abordam especificamente idosos e não está claro até que ponto elas podem ser utilizadas de maneira adequada nesses pacientes.^2 , 14 , 15^ A população idosa claramente beneficiou-se do progresso médico, com técnicas diagnóstico-terapêuticas que influenciam no aumento da expectativa de vida e em procedimentos menos invasivos.^10^ Um exemplo é o implante de válvula aórtica transcateter na abordagem de doenças da valva aórtica.^15^ No entanto, esses procedimentos, em conjunto com o implante crescente de dispositivos eletrônicos cardiovasculares (DEC), contribuem para infecções como a EI. Encontram-se comorbidades em mais da metade de idosos, com consequente necessidade de cuidados prolongados de profissionais da saúde, o que aumenta a probabilidade de aquisição da EI.^3 , 16^ O diagnóstico de EI em idosos, frequentemente, é atrasado ou esquecido.^3^ As manifestações podem ser inespecíficas, atribuídas ao envelhecimento e outras condições. A febre pode estar ausente, havendo apenas confusão mental.^17^ A EI pode se apresentar com complicações semelhantes a outras condições, como insuficiência cardíaca (IC), acidente vascular encefálico (AVE) ou embolia sistêmica atribuível a fibrilação atrial.^3 , 16^ No Brasil, apesar de uma população idosa crescente, até o momento não foi publicado nenhum artigo publicado a respeito de EI para esse grupo. O objetivo deste estudo é descrever o grupo de idosos em nossa coorte de EI em adultos e compará-los aos não idosos, ressaltando as diferenças entre os grupos.

## Métodos

O local do estudo é um hospital público terciário especializado em cardiologia de alta complexidade, com cirurgia cardíaca *in loco* . O estudo é retrospectivo de pacientes idosos definidos como pelo Estatuto do Idoso do Brasil,^18^ identificados na coorte de pacientes adultos com critério de EI definitiva pelos critérios modificados de Duke e conduzido pelo período de janeiro de 2006 a dezembro de 2019. As variáveis do estudo foram as incluídas em ficha de coleta de dados padrão ( *case report form* ) descritas previamente.^4^ A análise estatística foi realizada utilizando-se o programa Jamovi^®^, versão 1.2.2. Os dados foram expressos como frequências, médias ± desvio-padrão da média, mediana e intervalo interquartil. Para a análise bivariada, foram usados os testes do Qui-quadrado e Exato de Fisher. Para verificar a normalidade da distribuição, utilizou-se o teste de Shapiro-Wilk. Os testes t de Student não pareado e o de Mann-Whitney foram utilizados para comparar as variáveis numéricas entre os grupos de interesse. O valor de p < 0,05 foi considerado estatisticamente significante.

## Resultados

**Figura 1:**
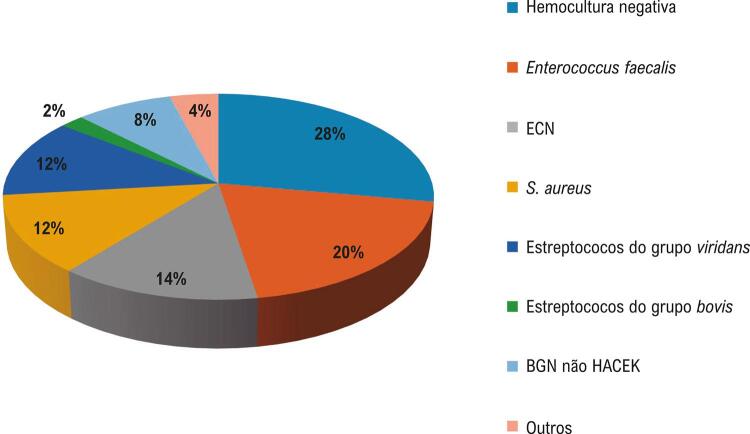
– *Agentes identificados em hemoculturas de 97 casos de EI em idosos (2006-2019). Outros: 1 Granulicatella; 1 Trichosporon beigelii; 1 Bartonella henselae; 1 Listeria monocytogenes. ECN: estafilococos coagulase-negativos; BGN: bastonetes Gram negativos; HACEK: Haemophilus spp, Aggregatibacter spp, Cardiobacterium hominis, Eikenella corrodens, Kingella kingae.*

**Figura 2:**
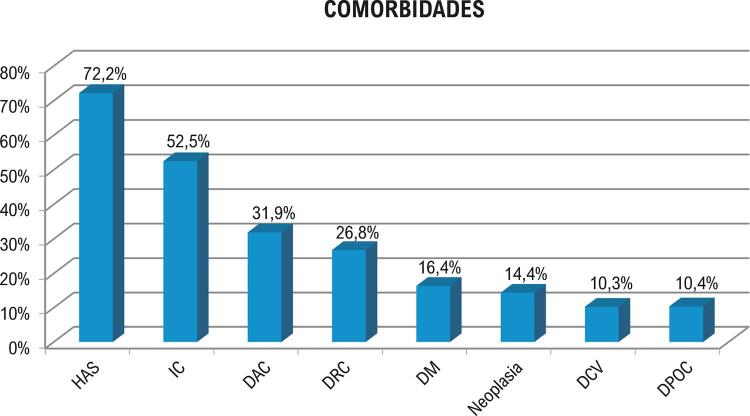
– *Comorbidades mais frequentes nos 97 idosos com EI (2006-2019). HAS: hipertensão arterial sistêmica; IC: insuficiência cardíaca; DAC: doença arterial coronariana; DRC: doença renal crônica; DM: diabetes melito; DCV: doença cerebrovascular; DPOC: doença pulmonar obstrutiva crônica.*

Idosos corresponderam a 97 de 370 casos de EI (26,2%) no período. A idade média foi 68,8±6,3 anos; sexo masculino correspondeu a 73 casos (75,2%). A apresentação foi aguda, isto é, sinais e sintomas foram observados em menos de 1 mês de evolução, em 60% dos casos (57/95) e subaguda em 40% (38/95). A aquisição foi comunitária em 49 (50,5%), nosocomial em 37 (38,1%) e relacionada a saúde não nosocomial em 11 (11,3%). Microrganismos mais prevalentes foram enterococos 18 (25,7%). Entre 12 *S. aureus* isolados, 10 (83,3%) eram MRSA e, desses, 6 eram hospitalares e 4 comunitários. Hemoculturas foram negativas em 27,8% ( Figura 1 ). Ecocardiograma transesofágico foi positivo em 88/96 (91,6%), e transtorácico em 75/96 (78,1%). Achados mais encontrados foram insuficiência aórtica em 37/96 (38,5%) e mitral em 43/96 (44,7%); vegetações aórticas em 40/96 (41,6%), mitrais em 36/96 (37,5%), tricúspides em 9/96 (9,3%), e em DEC em 11/96 (11,4%). As comorbidades mais frequentes foram hipertensão arterial, insuficiência cardíaca (IC) e doença arterial coronariana ( Figura 2 ); cirurgia cardíaca (CC) prévia foi relatada em 50/97 (51,5%). Havia predisposição de valva nativa em 36/92 (39,1%), prótese valvar em 45/97 (46,4%) e EI prévia em 10/97 (10,7%). As complicações foram IC por insuficiência aórtica ou mitral 57/97 (58,7%), abscesso 24/97 (24,7%), deiscência paravalvar protética 7/45 (15,5%), e perfuração valvar 25/97 (25,7%). Fenômenos embólicos esplênicos ocorreram em 28/97 (28,8%) e cerebrais em 18/97 (18,5%).

Dos 80 idosos com indicação cirúrgica (82,4%), 59 (73,7%) foram operados. A mortalidade hospitalar foi de 38 (39,1%); 22/59 (37%) morreram entre os que foram abordados cirurgicamente e 16/38 (42%) entre os que não foram.

Realizou-se uma análise comparativa utilizando dados de 359 pacientes adultos com EI de janeiro de 2006 a setembro de 2019 ( Tabela 1 ). Um total de 266 pacientes tinha idade < 60 anos, enquanto 93 pacientes (25,9%) tinha ≥ 60 anos. A proporção de homens entre idosos foi maior, assim como a evolução aguda e a EI hospitalar.

**Tabela 1:** – Comparação de características clínicas e laboratoriais, e desfechos entre idosos e não idosos com EI, realizada entre janeiro de 2006 e setembro de 2019

Variável/proporção em percentual (%)	Idosos(n=93)	Não idosos (n=266)	p valor
Gênero masculino	72	60,2	p = 0,04
Evolução aguda	63,3	46,8	p = 0,019
Aquisição hospitalar	39,8	24,5	p = 0,005
Aquisição relacionada a assistência à saúde não hospitalar	10,8	7,2	p = 0,285
Febre	88,2	94,7	p = 0,034
Sopro regurgitante novo	48,8	60,4	p = 0,064
Eventos embólicos	35,2	56,9	P < 0,001
Eventos embólicos para SNC	17,2	29,1	p = 0,025
Esplenomegalia	10,5	26,5	p = 0,002
Enterococos	20,4	7,5	p < 0,001
Estreptococos do grupo *viridans*	16	26	p = 0,047
Staphylococcus aureus	12,9	10,5	p = 0,531
Valvopatia reumática	20,2	37,7	0,002
Cardiopatia congênita	1,1	18	< 0,001
Prótese valvar*	25,7	11,8	0,006
HAS	72,9	39,2	< 0,001
DM	16,1	10,2	0,123
Dislipidemia	35,0	14,8	< 0,001
Fibrilação atrial	26,1	13,2	0,004
IC pregressa	53,3	35,7	0,003
DPOC	10,9	2,7	0,002
DAC	31,5	6,1	< 0,001
DCV	10,8	4,9	0,047
MP	20,4	8,3	0,002
IRC	27,5	17,7	0,044
Hemodiálise	7,5	7,1	0,902
Neoplasia	12,9	3,9	0,002
CC anterior	52,7	32,2	< 0,001
ICP	10,9	3,4	0,006
RVM	16,2	2,3	< 0,001
Uso de AAS	24,4	5,9	< 0,001
Uso de varfarina	24,4	18,1	0,222
Indicação cirúrgica para EI	81,7	88,7	p = 0,085
Ventilação mecânica**	30,3	18	p = 0,015
Uso de inotrópico**	33,7	21,8	p =0,028
Óbito intra-hospitalar	43,0	18,1	< 0,001

SNC: sistema nervoso central; HAS: hipertensão arterial sistêmica; DM: diabetes melito; IC: insuficiência cardíaca; DPOC: doença pulmonar obstrutiva crônica; DAC: doença arterial coronariana; DCA: doença cerebrovascular; MP: marcapasso; IRC: insuficiência renal crônica; CC: cirurgia cardíaca; ICP: intervenções cardíacas percutâneas; RVM: revascularização do miocárdio; AAS: ácido acetilsalicílico; * prótese valvar com mais de 1 ano de inserção; ** no pré-operatório de troca valvar para EI.

Quanto à clínica, pacientes idosos tiveram menos febre, sopro regurgitante novo, eventos embólicos, incluindo eventos para o sistema nervoso central, e menos esplenomegalia. Quanto a etiologia, idosos apresentaram enterococos com maior frequência, a estreptococos do grupo *viridans* em menor e frequência de *S. aureus* semelhante.

Idosos tiveram maior necessidade de ventilação mecânica e de inotrópicos antes da cirurgia. Não houve diferença quanto a insuficiência renal aguda, distúrbios de condução, embolização recorrente e abscessos. Não houve diferença na proporção de indicação cirúrgica entre idosos e NI. Idosos tiveram indicação cirúrgica em 81,7%, e foram operados em 66,3%; em comparação, dos 88,7% dos pacientes NI para os quais a cirurgia foi indicada, 84% foram operados. A indicação foi insuficiência ventricular secundária a regurgitação mitral ou aórtica aguda em 56,5% de idosos *versus* 63,4% de NI (p=0,243). Outras indicações cirúrgicas foram abscesso miocárdico/paravalvar em 21,5% de idosos *versus* 20% de NI (p=0,757); deiscência de prótese (6,5% *versus* 4,3%, respectivamente, p=0,409) e bacteremia persistente em 9% *versus* 4% de NI (p=0,062). A mortalidade foi mais que duas vezes maior em pacientes idosos ( Tabela 1 ).

## Discussão

Este estudo é inédito no Brasil, por ter enfoque na EI em idosos. Mais de um quarto dos pacientes com EI em nossa coorte de adultos eram idosos. Em estudos de países desenvolvidos, nota-se aumento da proporção de idosos entre casos de EI.^4 - 9^

Verificou-se frequência menor de febre, novo sopro regurgitante e complicações embólicas entre os idosos, o que foi constatado em outras publicações,^4 , 12^ esta última relacionada ao uso de antiagregantes plaquetários e/ou anticoagulantes. Tal situação pode indicar proteção para embolização com uso desses medicamentos; porém, são necessárias mais evidências científicas para comprovar essa hipótese. No estudo, idosos usaram significativamente mais ácido acetilsalicílico, e não varfarina, quando comparados aos NI.

Comorbidades foram mais prevalentes entre idosos, como esperado; tal fator é semelhante em estudo multicêntrico com grande número de pacientes, em que as frequências entre idosos e não idosos, respectivamente, de DM de 22,9% x 11,9% ( p < 0,001), de câncer geniturinário, de 4,7% x 0,6% ( p < 0,001) e de câncer do trato gastrintestinal de 3,2% x 0,8% (p < 0,001).^4^ Procedimentos invasivos prévios anteriores também foram mais frequentes entre idosos em nosso estudo, como foi nessa mesma publicação (56,2% x 38,5%, p < 0,001).^4^ Ratifica-se que a população idosa continua sendo mais exposta a procedimentos diagnósticos/terapêuticos, havendo maior predisposição à EI em função de eventos de bacteremia ocorridos nestes cenários e a presença de material sintético/dispositivos.

Observou-se maior frequência de EI hospitalar entre nossos idosos (39,8%); proporção semelhante é notada na literatura, em que a aquisição nosocomial representa 10,2% a 37% dos casos de EI em idosos.^6 , 11 , 12 , 19^ Na Tabela 2 , estão listados os estudos considerados mais relevantes de EI em idosos.

**Tabela 2:** – Aspectos da endocardite em idosos em uma revisão da literatura, 2000-2020

Autor	País	N^o^ do estudo	Período	Idade/ sexo	Valvas acometidas	Microrganismos mais frequentes	Condições subjacentes	Complicações	% Cirurgia	Mortalidade
Durante-Mangoni, 2008^4^	Vários	2.759	2000- 2005	1.056 idosos (65 anos ou mais); fem: (35,8%)	M (50%); Ao (41%); Tri (7%); dispositivos intracardíacos (10%); próteses (26%)	*S. aureus* (28,3%) (MRSA 35,8%); grupo *bovis* (8,3%); enterococos (16,5%); ECN (14%); estreptococos do grupo *viridans* (14,2%)	RM (63%); EA (28%); procedimento invasivo (56,2%); DM (22,9%); CA geniturinário (4,7%); GI (3,2%)	ICC (33,1%); AVE (14,6%); Embolia sistêmica (15,3%); Abscesso (14%); Bacteremia persistente (9,2%)	38,9%	24,9%
Remadi, 2009^17^	França	348	1991-2006	75 idosos (75 anos ou mais); masc: 47; fem: 28	M: 45,3%; Ao: 54,7%; M-Ao: 16%; EI lado direito: 16%; prótese valvar (28%); marcapasso (26,7%)	Estreptococos (37,4%); estafilococos (36 %); ECN (27,8%) em pacientes operados	DM 2 (25.3%); DRC (17,3%); neoplasia (26,7%)	ICC (28%); embolia 18,7%; evento maior de SNC 9,3%; Hemorragia Intracerebral 1,3% Abscessos 18,7%	29,3%	Geral: 16% Cirúrgica: 9%;
López, 2010^19^	Espanha	600 EI esquerda	1996-2008	Q3 (64-72 anos): 152; Q4 (>72 anos): 148	Nosocomial: 33%-37%; Prótese: 47%- 42%; prótese precoce: 36%- 39%; M(nat): 51%-61%; M(mec): 54%-35%; Ao (nat): 49%-39%; Ao (mec): 35%-21%; Ao (bio): 12%-33%	ECN: 22%-18%; *S. aureus*: 14%-14%; MRSA: 18%-33%; enterococos:12%-13%; grupo *viridans*: 12%-12%; grupo *bovis*: 5%-7%.	Reumáticos: 11%-8%; próteses 45- 48%; degeneração 12%-21%; DM: 28%- 29%; CA: 11%-13%; cateter IV: 6%-12%; C. cardíaca prévia: 14%- 12%	ICC: 59-64%; AVE: 18- 23% ; IRA: 40- 46%; Bacteremia Persistente: 32- 37%; Embolia: 24- 28%; Sepse: 14%- 16%; Abscesso perivalvar: 25- 26%.	54%-40%	Geral: 37%-36%; C. Urg: 44%-39%; C. Ele: 28%-34%. Trat. Med.: 40%-36%
Ramírez-Duque 2011^12^	Espanha	961 EI esquerda	1984-2008	65 anos ou mais: 356; Masc: 63,3%	Nosocomial: 21,1%; valva nativa: 74,5%; prótese tardia: 13,2%; prótese precoce: 12,4%; Ao: 50%; M: 37%; M-Ao: 11,4%;	Grupo *viridans*: 16.9%; *S. aureus*: 17.4%; MRSA 12.9%; ECN: 17,1%; enterococcos: 16,3%; grupo *bovis*: 5.3%; BGN: 4,2%	80%.	AVE: 26,6%; Embolia: 29,2%; IRA: 39,6%; sepse 16,5%; Complicações Intracardíacas: 27,8%; ICC: 28,9%	36%	Geral: 43,2%
Bassetti, 2014^5^	Itália	436	2004-2011	Grupo B (65-74 anos): 145; fem: 30%; grupo C (75 anos ou mais): 137; fem: 38,6%	Vegetação: 77,2%-78,1%	Enterococos: 11,7%-27%; estreptococos spp.: 33,1%-22,6%; *viridans*: 15,2%-7,3%; grupo *bovis*: 12,4%-11,2%; *S. aureus*: 14,5%-19,7%; ECN: 11%-13,1%	Doença valvar: 47,6%-40,2%; prótese: 40%-40,2%; ICC: 30,3%-47,7%; DRC: 22,1%-29,2%; DM2: 30,3%-23,4%; CA: 23,5%-25,6%; cardite reumática: 7,6%-2,9%	Embolia: 22,1%-13,9% Sinal neurológico focal: 11%-9,5%; Abscesso: 15,9%-14,6%	37,9%- 22,6%	Geral: 19,3%-22,6%.
Oliver, 2017^6^	França	454	2008-2013	G2 (65-80 anos): 173; Masc: 71,7%; G3 (acima de 80 anos): 51; Masc: 64,7%	Hosp.: 19,9%-23,5%; Pós-op..: 11%-11,8%; Valva nativa: 57,8%-58,8%; Ao G2: 42%; M G3: 43,1%; valva protética: 42,2%-41,2%; EI aguda: 57,1%-68,8%; EI > 3 meses: 14,3%-6,3%; principais portas de entrada (G3): TGI: 33,3%; TGU: 7,8%; pele (23,6%)	Enterococos: 15,6%-21,6%; Grupo *bovis*: 16,2% -17,7%; *viridans*: 17,9%-15,7%; ECN 9,3%-5,9%; MSSA: 12,7%-11,8%; MRSA: 2,9%- 2%	EI prévia: 11%-11,8%; DM2: 19,1%-25,5%; HAS: 49,1%-58,8%; DAC: 17,8%-17%; AVE: 9,3%-7,8%; DRC: 14,5%-27,5%; CA: 24,3%-29,4%	Embolia em ATB: G3: 21,6%; IRA: G3:51%; Espondilite: 12,6%-23,5% Abscesso: 26.5%-29,4%	69,6%-42,1%	Intrahospitalar: 13,3%-15,7%; Em 1 ano: 19,7%-37,3%; Cirúrgica: G3: 6,3%
Wu, 2019^11^	China	405	2007-2016	G3 (65 anos ou mais): 59; masc: 69,5%	Nativa: 83,1%; mitral: 25,4%; Ao: 30,5%; valvas à D: 6,8%; próteses: 13,6% marcapasso: 3,4%; comunitária: 79,7%; nosocomial: 10,2%	Estreptococos: 22%; *viridans*: 5,1%; estafilococos: 18,5%; *S. aureus:* 3,4%; ECN: 15,3%; enterococos: 1,7%	Reumáticos: 18,6%; cirurgia cardíaca prévia: 23,7%; degenerativa: 10,2%; DPOC: 3,4%; CA: 3,4%; HD: 6,8%; HAS: 42,4%; DM: 16,9%; Má higiene oral: 49,2%.	ICC: 62,7%; Embolia: 39%; IRA: 30,5%; AVE: 23,7%; Arritmia: 39% FA: 33,9%; Abscesso: 13,6%;	Indicação: 96,6% Cirurgia: 40,7%	Intra-hospitalar: 20,3%
Chun-Yu Lin, 2020^20^	Taiwan	179 EI esquerda	2005-2015	65 anos ou mais: 38; fem: 36,8%; média de idade: 74.2±6.4	Ao: 50% Troca por: Ao bio: 100%; Ao mec: 0%; M: 36,8 Troca por: M bio: 100%; M mec: 0%	Estreptococos: 28,9%; *Viridans*: 13,2%; Enterococos: 10,5%; *S. aureus*: 7,9%.	HAS: 13,2%; DM: 28,9%; DRC: 10,5%	VM: 23,7%; Inotrópicos: 10,5%; Embolia: 23,7%; IRA: 5,3%; FA: 13,2%; ICC: 18,4%; Abscesso: 2,6%	Todos os pacientes realizaram cirurgia, visto que o estudo avalia os pacientes submetidos à mesma	Intra-hospitalar: 26,3%

*Fem: feminino; masc: masculino; M: mitral; Ao: aórtica; Tri: tricúspide; MRSA: Meticilin Resistant Staphylococcus aureus; ECN: estafilococos coagulase negativo; RM: revascularização miocárdica; EA: estenose aórtica; DM: diabetes melito; CA: câncer; GI: gastrintestinal; ICC: insuficiência cardíaca congestiva; AVE: acidente vascular encefálico; EI: endocardite infecciosa; DRC: doença renal crônica; SNC: sistema nervoso central; (mec): mecânica; (nat): nativa; bio: biológica; IRA: injúria renal aguda; C. Urg: cirurgia de urgência; C. Ele: cirurgia eletiva; Trat. med.: tratamento medicamentoso; BGN: bacilos gram-negativos; P. valv.: procedimento valvar; hosp.: hospitalar; pós-op.: pós-operatória; TGU: trato geniturinário; HAS: hipertensão arterial sistêmica; DAC: doença arterial coronariana; DPOC: doença pulmonar obstrutiva crônica; VM: ventilação mecânica; FA: fibrilação atrial.*

Microrganismos mais prevalentes e observados em nossa série foram enterococos (25,7%), estreptococos do grupo *viridans* (17,1%) e *S. aureus* (17,1%). Ainda que estreptococos orais tenham sido anteriormente responsáveis pela maioria dos casos de EI em idosos, estafilococos predominam nas últimas décadas, especialmente *S. aureus* .^8 , 9^ Enterococos também estão relacionados a bacteremia por acessos vasculares, tendência epidemiológica que está ligada ao aumento da incidência de EI associada a cuidados de saúde.^5 , 6 , 12^ A frequência de EI causada por estreptococos que colonizam o trato digestivo, como *Streptococcus gallolyticus* e enterococos, acontece por conta da maior incidência de lesões do cólon em pacientes idosos.^4 , 8 , 19^ Todos os 7 pacientes com EI por grupo *bovis* tiveram o TGI investigado, mas não aqueles com EI por enterococos.

Em nosso centro de referencia em cirurgia cardíaca, houve indicação de troca valvar em mais de 4/5 dos idosos; porém, mais de 1/4 deles não foram operados. Tal fato possui aspecto multifatorial, incluindo idade avançada, múltiplas comorbidades, fragilidade, risco cirúrgico elevado e não aceitação de cirurgia pelo paciente ou sua família, entre outros, como observado através de estudo em EI em octogenários.^6^ Motivos pelos quais idosos que tiveram indicação cirúrgica não foram operados dizem respeito, sobretudo, ao *status* pré-operatório crítico, como observado pela elevada frequência de ventilação mecânica e uso de inotrópicos no pré-operatório. É importante notar que aneurismas micóticos e insuficiência renal aguda não foram mais frequentes entre idosos e NI, e que eventos de SNC foram menos frequentes em idosos. Em alguns estudos, a idade avançada é um preditor independente de mortalidade intra-hospitalar,^4 , 12^ o que influencia negativamente na decisão pelo procedimento. Contudo, em estudo recente realizado na China, notou-se que a sobrevida em 1 ano dos idosos que foram submetidos à abordagem cirúrgica foi maior do que nos indivíduos submetidos à terapêutica medicamentosa isoladamente (95,8% x 68,6%, p = 0,007).^11^ Além disso, mesmo entre os octogenários,^6^ os pacientes operados apresentam melhor sobrevida em 1 ano (93,6%) e em 3 anos (75,0%), respectivamente.

A mortalidade entre idosos em nosso estudo foi de 39,1%; na literatura, a taxa de óbitos variou de 16% a 43,2%.^4 - 6 , 11 , 12 , 17 , 19 , 20^ Por fim, notamos que:

Proporção expressiva (um quarto) das EI ocorreu em idosos, mesmo no sistema de saúde pública no Brasil;

Enterococos foi o patógeno mais frequente, e houve elevada proporção de MRSA entre a etiologia estafilocócica, o que sugere aquisição nosocomial ou foco gastrintestinal/geniturinário;

A clínica é menos exuberante em idosos, com menos febre, sopro novo e eventos embólicos;

A mortalidade nos idosos foi alta, o que sugere a contribuição da idade e das comorbidades, e possivelmente de diagnóstico tardio e não realização de cirurgia cardíaca.

## References

[1] Instituto Brasileiro de Geografia e Estatísticas. Estatísticas Sociais. Projeção da população 2018: número de habitantes do país deve parar de cresce em 2047 [Internet]. Rio de Janeiro: Agência de Notícias IBGE; c2021 [cited 2021 Aug 31]. Available from: https://agenciadenoticias.ibge.gov.br/agencia-sala-de-imprensa/2013-agencia-de-noticias/releases/21837-projecao-da-populacao-2018-numero-de-habitantes-do-pais-deve-parar-de-crescer-em-2047.

[2] Baddour LM, Wilson WR, Bayer AS, Fowler VG Jr, Tleyjeh IM, Rybak MJ, et al. Infective Endocarditis in Adults: Diagnosis, Antimicrobial Therapy, and Management of Complications: A Scientific Statement for Healthcare Professionals from the American Heart Association. Circulation. 2015;132(15):1435-86. doi: 10.1161/CIR.0000000000000296. 10.1161/CIR.0000000000000296 26373316

[3] Ursi MP, Durante-Mangoni E, Rajani R, Hancock J, Chambers JB, Prendergast B. Infective Endocarditis in the Elderly: Diagnostic and Treatment Options. Drugs Aging. 2019;36(2):115-24. doi: 10.1007/s40266-018-0614-7. 10.1007/s40266-018-0614-7 30488173

[4] Durante-Mangoni E, Bradley S, Selton-Suty C, Tripodi MF, Barsic B, Bouza E, et al. Current Features of Infective Endocarditis in Elderly Patients: Results of the International Collaboration on Endocarditis Prospective Cohort Study. Arch Intern Med. 2008;168(19):2095-103. doi: 10.1001/archinte.168.19.2095. 10.1001/archinte.168.19.2095 18955638

[5] Bassetti M, Venturini S, Crapis M, Ansaldi F, Orsi A, Della Mattia A, et al. Infective Endocarditis in Elderly: An Italian Prospective Multi-Center Observational Study. Int J Cardiol. 2014;177(2):636-8. doi: 10.1016/j.ijcard.2014.09.184. 10.1016/j.ijcard.2014.09.184 25449467

[6] Oliver L, Lavoute C, Giorgi R, Salaun E, Hubert S, Casalta JP, et al. Infective Endocarditis in Octogenarians. Heart. 2017;103(20):1602-9. doi: 10.1136/heartjnl-2016-310853. 10.1136/heartjnl-2016-310853 28432160

[7] Dhawan VK. Infective Endocarditis in Elderly Patients. Clin Infect Dis. 2002;34(6):806-12. doi: 10.1086/339045. 10.1086/339045 11830803

[8] Selton-Suty C, Célard M, Le Moing V, Doco-Lecompte T, Chirouze C, Iung B, et al. Preeminence of Staphylococcus Aureus in Infective Endocarditis: A 1-year Population-Based Survey. Clin Infect Dis. 2012;54(9):1230-9. doi: 10.1093/cid/cis199. 10.1093/cid/cis199 22492317

[9] Slipczuk L, Codolosa JN, Davila CD, Romero-Corral A, Yun J, Pressman GS, et al. Infective Endocarditis Epidemiology Over Five Decades: A Systematic Review. PLoS One. 2013;8(12):e82665. doi: 10.1371/journal.pone.0082665. 10.1371/journal.pone.0082665 PMC3857279 24349331

[10] Faulkner CM, Cox HL, Williamson JC. Unique Aspects of Antimicrobial Use in Older Adults. Clin Infect Dis. 2005;40(7):997-1004. doi: 10.1086/428125. 10.1086/428125 15824992

[11] Wu Z, Chen Y, Xiao T, Niu T, Shi Q, Xiao Y. The Clinical Features and Prognosis of Infective Endocarditis in the Elderly from 2007 to 2016 in a Tertiary Hospital in China. BMC Infect Dis. 2019;19(1):937. doi: 10.1186/s12879-019-4546-6. 10.1186/s12879-019-4546-6 PMC6836522 31694555

[12] Ramírez-Duque N, García-Cabrera E, Ivanova-Georgieva R, Noureddine M, Lomas JM, Hidalgo-Tenorio C, et al. Surgical Treatment for Infective Endocarditis in Elderly Patients. J Infect. 2011;63(2):131-8. doi: 10.1016/j.jinf.2011.05.021. 10.1016/j.jinf.2011.05.021 21679726

[13] Baddour LM, Epstein AE, Erickson CC, Knight BP, Levison ME, Lockhart PB, et al. Update on Cardiovascular Implantable Electronic Device Infections and their Management: A Scientific Statement from the American Heart Association. Circulation. 2010;121(3):458-77. doi: 10.1161/CIRCULATIONAHA.109.192665. 10.1161/CIRCULATIONAHA.109.192665 20048212

[14] Gould FK, Denning DW, Elliott TS, Foweraker J, Perry JD, Prendergast BD, et al. Guidelines for the Diagnosis and Antibiotic Treatment of Endocarditis in Adults: A Report of the Working Party of the British Society for Antimicrobial Chemotherapy. J Antimicrob Chemother. 2012;67(2):269-89. doi: 10.1093/jac/dkr450. 10.1093/jac/dkr450 22086858

[15] Habib G, Lancellotti P, Antunes MJ, Bongiorni MG, Casalta JP, Del Zotti F, et al. 2015 ESC Guidelines for the Management of Infective Endocarditis: The Task Force for the Management of Infective Endocarditis of the European Society of Cardiology (ESC). Endorsed by: European Association for Cardio-Thoracic Surgery (EACTS), the European Association of Nuclear Medicine (EANM). Eur Heart J. 2015;36(44):3075-128. doi: 10.1093/eurheartj/ehv319. 10.1093/eurheartj/ehv319 26320109

[16] Forestier E, Fraisse T, Roubaud-Baudron C, Selton-Suty C, Pagani L. Managing Infective Endocarditis in the Elderly: New Issues for an Old Disease. Clin Interv Aging. 2016;11:1199-206. doi: 10.2147/CIA.S101902. 10.2147/CIA.S101902 PMC5015881 27621607

[17] Remadi JP, Nadji G, Goissen T, Zomvuama NA, Sorel C, Tribouilloy C. Infective Endocarditis in Elderly Patients: Clinical Characteristics and Outcome. Eur J Cardiothorac Surg. 2009;35(1):123-9. doi: 10.1016/j.ejcts.2008.08.033. 10.1016/j.ejcts.2008.08.033 19062301

[18] Brasil. Secretaria Especial dos Direitos Humanos. Estatuto do Idoso: Lei Federal n^o^ 10.741. Brasília, DF (Oct 1 2003).

[19] López J, Revilla A, Vilacosta I, Sevilla T, Villacorta E, Sarriá C, et al. Age-Dependent Profile of Left-Sided Infective Endocarditis: A 3-Center Experience. Circulation. 2010;121(7):892-7. doi: 10.1161/CIRCULATIONAHA.109.877365. 10.1161/CIRCULATIONAHA.109.877365 20142448

[20] Lin CY, Lu CH, Lee HA, See LC, Wu MY, Han Y, et al. Elderly Versus Non-Elderly Patients Undergoing Surgery for Left-Sided Native Valve Infective Endocarditis: A 10-year Institutional Experience. Sci Rep. 2020;10(1):2690. doi: 10.1038/s41598-020-59657-1. 10.1038/s41598-020-59657-1 PMC7021775 32060394

