# Supplementary material for: Endocardite Infecciosa em Idosos: Características Distintas
Source: Arq Bras Cardiol. 2021 Oct 6;117(4):775–81. [Article in Portuguese] doi: 10.36660/abc.20201134 (PMC8528351; doi:10.36660/abc.20201134)
Supplement: Translation

# Translation: Infective Endocarditis in the Elderly: Distinct Characteristics

**Authors:** Luiz Henrique Braga Lemos, Leonardo Ribeiro da Silva, Marcelo Goulart Correa, Wilma Golebiovski, Clara Weksler, Rafael Quaresma Garrido, Giovanna Ferraiuoli Barbosa, Cristiane da Cruz Lamas

**Affiliations:** 1 Brazil Universidade do Grande Rio (Unigranrio), Rio de Janeiro, RJ – Brazil; 2 Brazil Instituto Nacional de Cardiologia, Rio de Janeiro, RJ – Brazil; 3 Brazil Instituto Nacional de Infectologia Evandro Chagas, Fiocruz, Rio de Janeiro, RJ – Brazil; 4 Brazil Universidade do Estado do Rio de Janeiro, Rio de Janeiro, RJ – Brazil

**Keywords:** Endocarditis, Aged, Comorbidity, Mortality, Thoracic Surgery

## Introduction

Infectious endocarditis (IE) is a serious disease with a mean in-hospital mortality of 20%.^1 - 3^ It has shown an increasing incidence, and the rise in its prevalence among the elderly population deserves to be highlighted.^4 - 9^ In elderly patients with IE, there are differences regarding the clinical presentation, complications, presence of comorbidities, therapeutic approach and mortality.^4 , 10 - 13^ IE guidelines do not specifically address the elderly population and it is not clear to what extent they can be appropriately used in these patients.^2 , 14 , 15^ The elderly population has clearly benefited from medical progress, with diagnostic-therapeutic techniques that influence the increase in life expectancy and less invasive procedures.^10^ One example is the transcatheter aortic valve implantation in the management of aortic valve diseases.^15^ However, these procedures, together with the increasing implantation of cardiovascular electronic devices (CED) contribute to infections such as IE. Comorbidities are found in more than half of the elderly, with the consequent need for long-term care from health professionals, which increases the probability of acquiring IE.^3 , 16^ The diagnosis of IE in the elderly is often delayed or forgotten.^3^ Manifestations may be nonspecific, attributed to aging and other conditions. Fever may be absent, with the presence of mental confusion only.^17^ IE can present with complications similar to those of other conditions, such as heart failure (HF), cerebrovascular accident (CVA) or systemic embolism attributable to atrial fibrillation.^3 , 16^ In Brazil, despite a growing elderly population, to date there is no published article about IE in this group. The aim of our study was to describe the elderly group in our adult IE cohort and compare them with non-elderly individuals, highlighting the differences between the groups.

## Methods

The study site is a tertiary, public, high-complexity cardiology hospital with on-site cardiac surgery. This is a retrospective study of elderly patients, as defined by the Statute of the Elderly of Brazil,^18^ identified in the cohort of adult patients with definitive IE, according to the modified Duke criteria, conducted from January 2006 to December 2019. The study variables were included in the previously described data collection form (case report form).^4^ The statistical analysis was performed using the Jamovi software, version 1.2.2. Data were expressed as frequencies, means ± standard deviation of the mean, median and interquartile range. For the bivariate analysis, Chi-square and Fisher’s exact tests were used. The Shapiro-Wilk test was used to verify the normality of the distribution. The unpaired Student’s *t* test and the Mann-Whitney test were used to compare numerical variables between the groups of interest. A value of p<0.05 was considered statistically significant.

## Results

Elderly patients accounted for 97 of 370 (26.2%) cases of IE in the period. The mean age was 68.8±6.3 years; the male gender corresponded to 73 cases (75.2%). The presentation was acute, i.e., signs and symptoms were observed in less than one month of evolution, in 60% (57/95) of the cases and subacute in 40% (38/95). The disease was community acquired in 49 (50.5%), nosocomial in 37 (38.1%) and related to non-nosocomial health care in 11 (11.3%). The most prevalent microorganisms were *enterococci* 18 (25.7%). Of the 12 isolated *S. aureus* cases, 10 (83.3%) were MRSA and of these, 6 were hospital and 4 community-acquired cases. Blood cultures were negative in 27.8% ( Figure 1 ). Transesophageal echocardiography was positive in 88/96 (91.6%), and transthoracic echocardiography in 75/96 (78.1%). The most common findings were aortic regurgitation in 37/96 (38.5%) and mitral regurgitation in 43/96 (44.7%); aortic vegetation in 40/96 (41.6%), mitral in 36/96 (37.5%), tricuspid in 9/96 (9.3%), and in CED in 11/96 (11.4%). The most frequent comorbidities were arterial hypertension, heart failure (HF) and coronary artery disease ( Figure 2 ); previous heart surgery (HS) was reported in 50/97 (51.5%). There was predisposition of the native valve in 36/92 (39.1%), prosthetic valve in 45/97 (46.4%) and previous IE in 10/97 (10.7%). The complications were HF due to aortic or mitral regurgitation 57/97 (58.7%), abscess 24/97 (24.7%), prosthetic paravalvular dehiscence 7/45 (15.5%), and valve perforation in 25/97 (25.7%). Splenic embolic phenomena occurred in 28/97 (28.8%) and cerebral in 18/97 (18.5%).

**Figure 1:** – *Agents identified in blood cultures of 97 cases of IE in the elderly, 2006-2019. Others: 1 Granulicatella, 1 Trichosporon beigelii, 1 Bartonella henselae, 1 Listeria monocytogenes. CNS: coagulase-negative staphylococci; GNB: Gram negative bacilli; HACEK: Haemophilus spp, Aggregatibacter spp, Cardiobacterium hominis, Eikenella corrodens, Kingella kingae.*

**Figure 2:** – *Most frequent comorbidities in 97 elderly people with IE, 2006-2019. SAH: Systemic Arterial Hypertension; HF: Heart Failure; CAD: Coronary Artery Disease; CKD: Chronic Kidney Disease; DM: Diabetes Mellitus; CVD: Cerebrovascular Disease; COPD: Chronic Obstructive Pulmonary Disease.*

Of the 80 (82.4%) elderly with surgical indication, 59 (73.7%) were submitted to surgery. Hospital mortality was 38 (39.1%); 22/59 (37%) died among those who were submitted to surgery and 16/38 (42%) among those who were not.

A comparative analysis was performed using data from 359 adult patients with IE from January 2006 to September 2019 ( Table 1 ). A total of 266 patients were aged <60 years, while 93 (25.9%) patients were aged ≥60 years. The proportion of men among the elderly was higher, as well as the acute evolution and in-hospital IE.

**Table 1:** – Comparison of clinical-laboratory characteristics and outcomes between elderly and non-elderly individuals with IE, January 2006 to September 2019

Variable/proportion in percentage (%)	Elderly (n=93)	Non-elderly (n=266)	p-value
Male gender	72	60.2	p=0.04
Acute evolution	63.3	46.8	p=0.019
Hospital-acquired infection	39.8	24.5	p=0.005
Non-hospital healthcare setting acquired infection	10.8	7.2	p=0.285
Fever	88.2	94.7	p=0.034
New regurgitating murmur	48.8	60.4	p=0.064
Embolic events	35.2	56.9	p<0.001
Embolic events for CNS	17.2	29.1	p=0.025
Splenomegaly	10.5	26.5	p=0.002
Enterococci	20.4	7.5	p<0.001
Viridans group streptococci	16	26	p=0.047
Staphylococcus aureus	12.9	10.5	p=0.531
Rheumatic valve disease	20.2	37.7	0.002
Congenital cardiopathy	1.1	18	<0.001
Valve prosthesis*	25.7	11.8	0.006
SAH	72.9	39.2	<0.001
DM	16.1	10.2	0.123
Dyslipidemia	35.0	14.8	<0.001
Atrial fibrillation	26.1	13.2	0.004
Previous HF	53.3	35.7	0.003
COPD	10.9	2.7	0.002
CAD	31.5	6.1	<0.001
CVD	10.8	4.9	0.047
PM	20.4	8.3	0.002
CKD	27.5	17.7	0.044
Hemodialysis	7.5	7.1	0.902
Neoplasia	12.9	3.9	0.002
Previous HS	52.7	32.2	<0.001
PCI	10.9	3.4	0.006
CABG	16.2	2.3	<0.001
ASA use	24.4	5.9	<0.001
Warfarin use	24.4	18.1	0.222
Surgical indication for IE	81.7	88.7	p=0.085
Mechanical ventilation**	30.3	18	p=0.015
Inotropic use**	33.7	21.8	p=0.028
In-hospital death	43.0	18.1	<0.001

CNS: central nervous system; SAH: systemic arterial hypertension; DM:diabetes mellitus; HF: heart failure; COPD: chronic obstructive pulmonary disease; CAD: coronary artery disease; CVD: cerebrovascular disease; PM: pacemaker; CKD: chronic kidney disease; HS: heart surgery; PCI: percutaneous coronary interventions; CABG: coronary artery bypass graft; ASA: acetylsalicylic acid; *valve prosthesis with more than 1 year of insertion; ** in the preoperative period of valve replacement for IE.

Regarding clinical features, elderly patients had less often presented with fever, new regurgitant murmur, embolic events, including central nervous system events, and splenomegaly. As for the etiology, the elderly had *enterococci* more frequently, *streptococci* from the *Viridans* group less frequently, and similar frequency of *S. aureus* . Elderly individuals had a greater need for mechanical ventilation and inotropic agent use before the surgery. There was no difference regarding acute kidney injury, conduction disorders, recurrent embolization and abscesses. There was no difference regarding the proportion of surgical indications between the elderly and non-elderly (NE). Elderly individuals had surgical indication in 81.7% of the cases and 66.3% were submitted to surgery; in comparison, of the 88.7% of NE patients for whom surgery was indicated, 84% were operated. The indication was heart failure failure secondary to acute mitral or aortic regurgitation in 56.5% of the elderly vs. 63.4% of NE (p=0.243). Other surgical indications were myocardial/paravalvular abscess in 21.5% of elderly vs. 20% of NE (p=0.757); prosthesis dehiscence (6.5% vs. 4.3%, respectively, p=0.409) and persistent bacteremia in 9% vs. 4% of NE (p=0.062). Mortality was more than twice as high in elderly patients ( Table 1 ).

## Discussion

Our study is an unprecedented one in Brazil, as it focuses on IE in the elderly. More than a quarter of the patients with IE in our cohort of adults were elderly, and studies from developed countries have shown an increase in the proportion of elderly individuals among IE cases.^4 - 9^ A lower frequency of fever, new regurgitant murmur and embolic complications was identified among the elderly, which was found in other publications,^4 , 12^ with the latter being related to the use of antiplatelet and/or anticoagulant agents. This situation may indicate protection against embolization with the use of these drugs, but more scientific evidence is required to prove this hypothesis. In our study, elderly individuals used significantly more aspirin, but not warfarin, when compared to NE.

Comorbidities were more prevalent among the elderly, as expected; this is similar to a multicenter study with a large number of patients, with the frequencies in the elderly and non-elderly, of DM being 22.9% vs. 11.9% (p<0.001); of genitourinary cancer, being 4.7%, respectively, vs. 0.6% (p<0.001) and 3.2% vs. 0.8% of gastrointestinal tract cancer (p<0.001).^4^ Previous invasive procedures were also more frequent among the elderly in our study, as it was in this same publication (56.2% vs. 38.5%, p<0.001).^4^ It has been confirmed that the elderly population continues to be more exposed to diagnostic/therapeutic procedures, with a greater predisposition to IE due to bacteremic events that occurred in these scenarios and the presence of synthetic material/devices.

A higher frequency of in-hospital IE was observed, which represented 39.8% among our elderly patients; a similar proportion has been observed in the literature, in which hospital-acquired disease represents 10.2 to 37% of IE cases in the elderly.^6 , 11 , 12 , 19^Table 2 lists the IE studies carried out in the elderly that are considered to be the most relevant ones.

**Table 2:** – Aspects of endocarditis in the elderly in a literature review, 2000-2020

Author	Country	N of study	Period	Age/ Sex	Affected valves	More frequent microorganisms	Underlying conditions	Complications	% of Surgeries	Mortality
Durante-Mangoni, 2008^4^	Several	2,759	2000- 2005.	1056 elderly (65 years and over) females: (35.8%)	Mi (50%) Ao (41%) Tri (7%) Intracardiac devices (10%); prostheses (26%)	*S. aureus* (28.3%) (MRSA 35.8%), *bovis* group (8.3%), *Enterococci* (16.5%) CoNS (14%); *Viridans* group *streptococci* (14.2%)	CABG (63%) AS (28%) invasive procedure (56.2%) DM (22.9%) and GUT (4.7%) and GIT CA (3.2%)	CHF (33,1%); CVA (146%); systemic embolism (15.3%); Abscess (14%); persistent Bacteremia (9.2%)	38.9%	24.9%
Remadi, 2009^17^	France	348	1991 – 2006	75 elderly (75 years or older) M:47; F:28	Mi: 45.3%; Ao: 54.7%; Mi-Ao: 16%; right-side IE: 16% valvar prosthesis (28%); Pacemaker (26.7%)	*Streptococci* (37.4%), *staphylococci* (36 %). CoNS (27.8%) in patients submitted to surgery.	DM 2 (25.3%); CKD (17.3%); Neoplasia (26.7%);	CHF (28%); embolism 18.7%; CNS major event 9,3%; Intracerebral Hemorrhage 1.3% Abscesses 18.7%	29.3%	Overall: 16% Surgical: 9%;
Lopez, 2010^19^	Spain	600 Left-side IE cases	1996-2008	Q3 (64-72 years): 152 and Q4 (>72 years): 148.	Nosocomial: 33%-37%; Prosthesis: 47%- 42%; Early prosthesis: 36%- 39%; Mi (nat): 51%-61% Mi (mech): 54%-35% Ao (nat): 49%-39% Ao (mech): 35%-21% Ao (bio): 12%-33%	CoNS: 22%-18%. *S. aureus*: 14%-14% MRSA: 18%-33%. *Enterococci*: 12%-13%; *Viridans* group: 12%-12% *bovis* group: 5%-7%.	Rheumatics: 11%-8%. Prostheses 45- 48%; Degeneration 12%-21%; DM: 28%- 29%. CA: 11%-13%. IV Catheter: 6%-12% Previous H. surgery: 14%- 12%.	CHF : 59-64% ; CVA: 18- 23% ; AKI: 40- 46%; Persistent Bacteremia: 32- 37%; Embolism: 24- 28%; Sepsis: 14%- 16%; Perivalvar abscess: 25- 26%.	54%-40%	Overall: 37%-36%; Urg. S: 44%-39%; Elec S.: 28%-34%. D. Treatment: 40%-36%
Ramírez-Duque 2011^12^	Spain	961 Left IE cases	1984 –2008	65 years or older: 356 M: 63.3%	Nosocomial: 21.1%; native valve: 74,5%; Late prosthesis: 13.2%; Early prosthesis: 12.4% Ao: 50%; Mi: 37%; Mi-Ao: 11.4%;	*Viridans* group: 16.9%; *S. aureus*: 17.4%; MRSA 12.9%; CoNS: 17.1%; *Enterococci*: 16.3%; *bovis* group: 5.3%; GNB: 4.2%.	80%.	CVA: 26.6%; Embolism: 29,2%; AKI: 39.6%; sepsis 16.5%; Intracardiac Complications: 27.8%; CHF: 28.9%	36%	Overall: 43.2%
Bassetti, 2014^5^	Italy	436	2004 –2011	Group B: 65-74 years: 145 F: 30% Group C: 75 years or older: 137 F: 38.6%	Vegetation: 77.2%-78.1%	*Enterococci*: 11.7%-27%; *Streptococci spp* .: 33.1%-22.6%; *Viridans* group: 15.2%-7.3%; *bovis* group: 12.4%-11.2%; *S. aureus*: 14.5%-19.7%; CoNS: 11%-13.1%.	Valve disease: 47.6%-40.2%; Prosthesis: 40%-40.2% CHF: 30.3%-47.7%; CKD: 22.1%-29.2% DM2: 30.3%-23.4% CA: 23.5%-25.6% rheumatic carditis: 7.6%-2.9%	Embolism: 22.1%-13.9% Focal neurological sign11%-9.5%; Abscess: 15.9%-14.6%	37.9%- 22.6%	Overall: 19.3%-22.6%.
Oliver, 2017^6^	France	454	2008 –2013	G2: 65-80 years: 173 M: 71.7% G3: age > 80 years: 51 M: 64.7%	Hosp.: 19.9%-23.5%; Post-op.: 11%-11.8%; Native valve: 57.8%-58.8%; Ao: G2: 42% Mi G3: 43.1%; Prosthetic valve: 42.2%-41.2%; Acute IE: 57.1%-68.8%; IE > 3 months: 14.3%-6.3%; Most common sites of entry (G3): GIT: 33.3%, GUT: 7.8%, skin (23.6%);	*Enterococci*: 15.6%-21.6% ; *bovis* group: 16.2% - 17.7%; *viridans* group: 17.9%-15.7%; CoNS 9,3%-5.9%; MSSA: 12.7%-11.8%; MRSA: 2.9%- 2%	Previous IE: 11%-11.8%; DM2: 19.1%-25.5%; SAH: 49.1%-58.8%; CAD: 17.8%-17%; CVA: 9.3%-7.8%; CKD: 14.5%-27.5%; CA: 24.3%-29.4%	Embolism in ATB: G3: 21.6%; AKI: G3:51%; Spondylitis: 12.6%-23.5% Abscess: 26.5%-29.4%	69,6%-42,1%	In-hospital: 13.3%-15.7%; One-year: 19.7%-37.3%; Surgical: G3: 6,3%
Wu, 2019^11^	China	405	2007 – 2016	G3: 65 years or older: 59 M: 69.5%	Native: 83.1%; Mitral: 25.4%; Ao: 30.5%; Valves to the right: 6.8%; Prostheses: 13.6% Pacemaker: 3.4%; Community: 79.7%; Nosocomial: 10.2%	*Streptococci*: 22%; *Viridans* group: 5.1%; *Staphylococci*: 18.5%; *S. aureus:* 3.4%; CoNS: 15.3%; *Enterococci*: 1.7%.	Rheumatics: 18.6%; Previous Heart Surgery: 23.7%; Degenerative: 10.2%; COPD: 3.4%; CA: 3.4%; HD: 6.8%; SAH: 42.4%; DM: 16.9%; Bad oral hygiene: 49.2%.	CHF: 62.7%; Embolism: 39%; AKI: 30.5%; CVA: 23.7%; Arrythmia: 39% AF: 33.9%; Abscess: 13.6%;	Indication: 96.6% Surgery: 40.7%	In-hospital: 20.3%
Chun-Yu Lin, 2020^20^	Taiwan	179 left IE cases	2005 –2015	65 years or older: 38 F: 36.8% Mean age: 74.2±6.4	Ao: 50% Exchange for: bio Ao: 100%; mech Ao: 0% Mi: 36.8 Exchange for: bio Mi: 100%; mech Mi: 0%.	*Streptococci*: 28.9%; *Viridan* s group: 13.2%; *Enterococci*: 10.5%; *S. aureus*: 7.9%.	SAH: 13.2%; DM: 28.9%; CKD: 10.5%;	MV: 23.7%; Inotropic drugs: 10.5%; Embolism: 23.7%; AKI: 5.3%; AF: 13.2%; CHF: 18.4%; Abscess: 2.6%	All patients underwent surgery, as the study evaluates patients undergoing it	In-hospital: 26.3%

*F: female; M: male; Mi: mitral; Ao: aortic; Tri: tricuspid; MRSA: methicillin-resistant Staphylococcus aureus; CoNS: Coagulase Negative Staphylococci; CABG: coronary artery bypass graft; AS: aortic stenosis; DM: diabetes mellitus; CA: cancer; GIT: gastrointestinal tract; CHF: congestive heart failure; CVA: cerebrovascular accident; IE: infective endocarditis; CKD: chronic kidney disease; CNS: central nervous system; (mech): mechanical; (nat): native; (bio): biological; AKI: acute kidney injury; Urg. S: urgent surgery; Elec. S: elective surgery; D. Treatment: Drug Treatment; GNB: Gram-Negative Bacilli; Valv. P.: Valve procedure; Hosp.: Hospital; Post-op.: Post-operative; GUT: genitourinary tract; SAH: systemic arterial hypertension; CAD: coronary artery disease; COPD: chronic obstructive pulmonary disease; MV: mechanical ventilation; AF: atrial fibrillation.*

The most prevalent microorganisms observed in our series were *enterococci* (25.7%), *streptococci* from the *Viridans* group (17.1%) and *S. aureus* (17.1%). Although oral *streptococci* have been previously responsible for most cases of IE in the elderly, *staphylococci* have predominated in recent decades, especially *S. aureus.* ^8 , 9^ *Enterococci* are also related to bacteremia caused by vascular access. This epidemiological trend is linked to the increased incidence of IE associated with healthcare.^5 , 6 , 12^ The frequency of IE caused by *streptococci* that colonize the digestive tract, such as *Streptococcus gallolyticus* and *enterococci* , is also higher due to the higher incidence of colon lesions in elderly patients;^4 , 8 , 19^ all 7 patients with IE caused by the *bovis* group had their GIT investigated, but not those with IE caused by *enterococci* .

In our referral center for heart surgery, valve replacement was indicated in more than 4/5 of the elderly, but more than 1/4 of them were not submitted to surgery. This fact has a multifactorial aspect, including older age, multiple comorbidities, frailty, high surgical risk, non-acceptance of surgery by the patient or their family, among others, as observed in a study on IE in octogenarians.^6^ In our study, the reasons why elderly individuals who had surgical indication were not submitted to the surgical procedure are mainly related to their critical preoperative status, as noted by the high frequency of mechanical ventilation and the use of inotropic agents in the preoperative period. It is noteworthy that events such as mycotic aneurysms and acute kidney injury were not more frequent among the elderly compared to the NE, and that CNS events were less frequent in the elderly. In some studies, older age is an independent predictor of in-hospital mortality,^4 , 12^ which negatively influences the decision to perform the procedure. However, in a study carried out recently in China, it was observed that the one-year survival among the elderly submitted to surgical procedures was greater than that of those who underwent drug therapy alone (95.8% vs. 68.6%, p = 0.007).^11^ Moreover, even among octogenarians,^6^ those submitted to the surgical procedure showed better one-year (93.6%) and three-year (75.0%) survival, respectively. Mortality among the elderly in our study was 39.1%; in the literature, the mortality varies from 16% to 43.2%.^4 - 6 , 11 , 12 , 17 , 19 , 20^

As conclusions, we observed that i) a significant proportion (one quarter) of IE occurred in the elderly, even in the public health system in Brazil ii) *enterococci* were the most frequent pathogens, and there was a high proportion of MRSA in the *staphylococcal* etiology, suggesting nosocomial acquisition or gastrointestinal/genitourinary focus, iii) the clinical picture is less exuberant in the elderly, with less fever, new murmur and embolic events and iii) mortality in the elderly was high, suggesting the contribution of age and comorbidities, and possibly a late diagnosis and not undergoing cardiac surgery.
